# Methylation Status of MTHFR Promoter and Oligozoospermia Risk: An Epigenetic Study and in Silico Analysis

**DOI:** 10.22074/cellj.2021.6498

**Published:** 2020-04-22

**Authors:** Atefeh Rezaeian, Mohammad Karimian, Abasalt Hossienzadeh Colagar

**Affiliations:** 1 Department of Cellular Biotechnology, Cell Science Research Center, Royan Institute for Biotechnology, ACECR, Isfahan, Iran; 2 Department of Molecular and Cell Biology, Faculty of Basic Sciences, University of Mazandaran, Babolsar, Iran

**Keywords:** Bioinformatics, DNA Methylation, Male Infertility, Methylenetetrahydrofolate Reductase, Oligozoospermia

## Abstract

**Objective:**

In this study, we evaluated the effects of promoter methylation of MTHFR on oligozoospermia risk, followed by an in silico analysis.

**Materials and Methods:**

In a case-control study, semen samples were collected from infertile and healthy control men. MTHFR promoter region was amplified by methylation-specific polymerase chain reaction (PCR). Finally, the promoter region of MTHFR was analyzed by bioinformatics software.

**Results:**

Our data revealed significant associations of CpG island promoter methylation with oligozoospermia in a case-control study. In silico analysis showed that promoter contains a strong nucleosome exclusion region, a bonafide CGIs, six PROSITE motifs without a defined TATA box and 14 transcription factor (TF) binding sites, which are directly involved in spermatogenesis

**Conclusion:**

Based on our findings, methylation of the MTHFR gene promoter region may be a risk factor for oligozoospermia. However, this is a preliminary report representing data for future comprehensive studies to make a clinical conclusion on the potential biomarker role of methylation of this promoter in elevating susceptibility to oligozoospermia.

## Introduction

Human infertility is a major health problem in 10-15% of couples worldwide. Male factors are responsible for 50% of infertility causes (1). There are many environmental, genetic and epigenetic risk factors for male infertilities. About 15-30% of the male infertilities are due to genetic abnormalities, such as chromosomal or monogenic disorders, mitochondrial DNA (mtDNA) mutations, micro-deletions on Y chromosome and autosomal deletions, defects in DNA repair mechanism, Y-linked syndromes and some single nucleotide polymorphisms (SNPs). In addition to the genetic factors, epigenetics may also affect male infertility (2-4). Epigenetics refers to the heritable alteration in gene expression and activity without any change of DNA sequences (5). Epigenetic modifications such as acetylation, de-acetylation, methylation and de-methylation of DNA and proteins lead to different patterns of gene expression (6).

Epigenetics play a crucial role in development and function of sperm, fertilization and post-fertilization events (5). DNA methylation is the most common epigenetic factor which may affect male reproduction (7). DNA methylation occurs by addition of a methyl group in the fifth position of the cytosine ring among the CpG (cytosine-phosphate-guanine) di-nucleotides (8). Hyper-methylation of CpG rich sequences (called CpG islands) in promoter region usually leads to down-regulation, whereas hypo-methylation generally leads to up-regulation of genes (9).

One of the key enzymes involved in spermatogenesis process is methylene tetrahydrofolate reductase (MTHFR; OMIM: 607093). MTHFR converts 5, 10-methylenetetrahydrofolate to 5-methyltetrahydrofolate, which donates a methyl group for re-methylation of homocysteine to methionine. Then, methionine provides the methyl group for S-adenosylmethionine, a major methyl group donor for various reactions such as DNA, RNA and protein methylation. On the other hand, 5, 10-methylenetetrahydrofolate is an essential substrate for thymidylate synthase. Therefore, MTHFR is a key enzyme regulating methylation reactions and nucleic acid synthase (10).

Alteration of *MTHFR* gene expression may also influence spermatogenesis process. *MTHFR* transcript level is lower in human tissues, compared to testis, showing that MTHFR has a critical role in germ cell maturation (11). High levels of MTHFR in testis ensures that sufficient amounts of folate derivatives move toward production of methyl group -S adenosyl methionine, as the main donor of methyl group in the methylation reaction. Therefore, alterations in the methyl supplies, raised from *MTHFR* insufficiency, may influence spermatogenesis (12). However, methylation patterns are amazingly similar among the embryonic stem cells, embryonic germ cells and sperms (13). Promoter methylation of this gene may influence the expression of MTHFR and subsequently male infertility (14).

The human *MTHFR* gene consists of 12 exons and is located on 1p36.3 (10). There is no CAAT and TATA-box elements in the promoter of this gene, but it has multiple binding sites for transcription factors (TFs) including SP1, HINF-3, NF-GMa, c-Rel, UTRF, E2-F1, NF-kB and AP1 (11). Here we investigated promoter methylation in semen samples with reduced sperm count and fertile men, to evaluate its role in oligozoospermia. In addition, the bioinformatics approach was applied to validate the laboratory results.

## Materials and Methods

### Subjects and sample collection

In this case-control study, semen samples were collected from 151 subjects (including 73 oligozoospermic men with age of 36.28 ± 3.49 years and 78 fertile men with age of 37.42 ± 4.11 years). The infertile men who referred to Shahid Beheshti Infertility Center (Kashan, Iran) had no history of cryptorchidism, orchitis, infectious disease, diabetes mellitus, drug abuse, obstruction of the vas deferens, varicocele, abnormal profiles of luteinizing hormone (LH), follicle-stimulating hormone (FSH) and testosterone, abnormal karyotype as well as Y-chromosome microdeletion. In addition, the infertile men’s women had no problem in their reproductive systems. The control group was comprised of volunteer men with no history of infertility and at least one healthy offspring who were referred to the same clinic for sperm analysis to approve normal sperm parameters. Semen parameters were analyzed according to World Health Organization, 2010 criteria (15). Therefore, men with less than 15 million/ml sperm count, less than 39% progressive motility, and less than 4% normal form were classified as oligozoospermic, asthenozoospermic and teratozoospermic, respectively. In this study, all of the infertile men were identified as oligozoospermic. Finally, semen samples were collected from all subjects into sterile tubes. Informed written consent was obtained from all of the participants and this study was confirmed by the principles outlined in the Declaration of Helsinki and approved by the Medical Ethics Committee of Kashan University of Medical Sciences (Kashan, Iran, IR.KAUMS.REC.1396.24).

### Sperm preparation, DNA isolation and methylationspecific polymerase chain reaction

**Fig.1:**
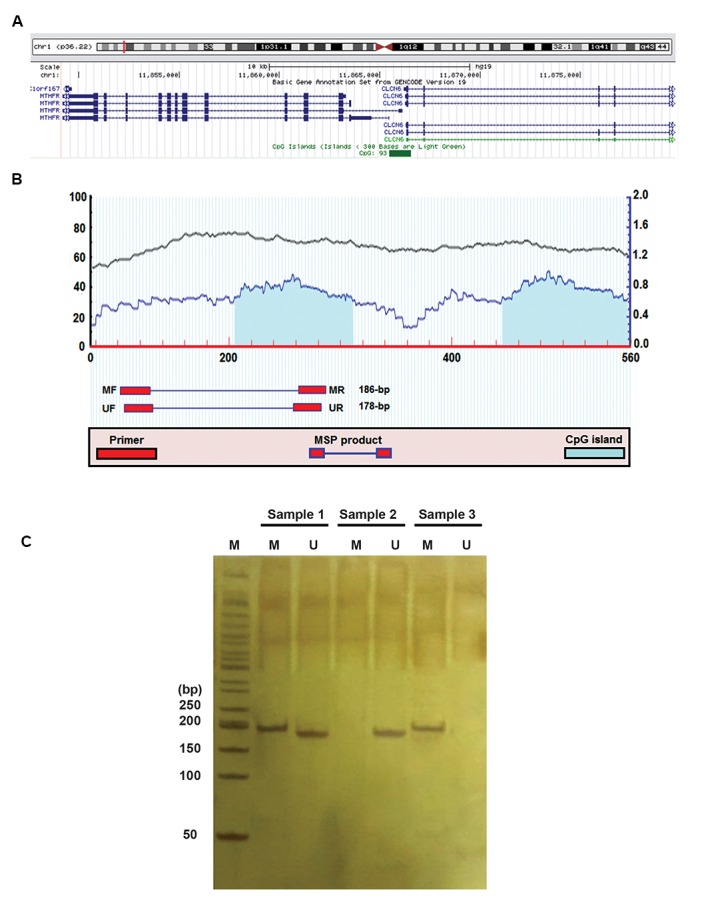
CpG map of *MTHFR* and methylation-specific polymerase chain reaction (PCR) result. **A.** *MTHFR* gene is located on chromosome 1 (1p36.22), the region containing CpG islands demonstrated by the green box.** B.** MethPrimer software indicated presence of two CpG islands in *MTHFR* promoter. The location of methylated and unmethylated primers as well as the PCR products was illustrated. Length of methylated and unmethylated PCR products respectively shows 186 and 178 bp. **C. **MS-PCR results showed the heterogeneous samples amplifying both methylated and unmethylated primers (sample 1), while the unmethylated (sample 2) and methylated (sample 3) samples were just amplified with unmethylated and methylated primers, respectively.

An osmotic shock process was employed to avoid sperm contamination with some other cells such as lymphocytes and epithelial cells. To remove the extra cells, sperm mixture was treated with Tris-HCl (50 mM, pH=6.8) at 8˚C for 20 minutes and then the mixture was centrifuged to collect the purified spermatozoa. Genomic DNA was extracted from semen samples with DNG plus DNA extraction buffer (Cinnagen, Iran). We analyzed a single gene promoter in our study. CpG methylation in the promoter region of the *MTHFR* gene was detected by methylation specific PCR (MSP, also known as MS-PCR) method. For this purpose, the entire sequence of *MTHFR* gene was deduced from NCBI database (Accession no. NG_013351.1). The specific primers for methylated and unmethylated forms of the promoter region were designed by MethPrimer online software (16). The promoter region of *MTHFR* was analyzed by the mentioned software, showing two CpG islands in that region. As depicted in Figure 1, the specific methylated and unmethylated primers were designed on the upstream CpG island. DNA samples were treated with sodium bisulfite using EpiTect Bisulfite Kit (Qiagen, USA) which converts unmethylated cytosine residues to uracil, while it has no effect on the methylated cytosine. Treated DNA was purified by EpiTect spin columns of the aforementioned Kit. MS-PCR was carried out for methylated and unmethylated primers separately in 12.5 μl PCR mixture containing 30 ng of treated DNA, 0.1 ml Taq DNA polymerase, 0.12 ml dNTPs mix, 0.17 μM forward primer, 0.17 μM reverse primer and 1.5 mM MgCl_2_. PCR was performed in an Eppendorf thermal cycler (Mastercycler, Eppendorf, Germany). All of the PCR reagents were ordered from Cinnagen Company. Finally, the amplified fragments were separated by electrophoresis on 8% polyacrylamide gel electrophoresis and stained with silver nitrate (AgNO_3_; Sigma Aldrich, UK). SssI methyltransferase (New England Biolabs, USA) was applied for methylation of one sperm DNA sample as the methylated positive control.

### Statistical analysis

A Chi-square test was used to evaluate the differences in frequency of methylated, unmethylated and heterogeneous samples between case and control groups. A binary regression logistic was applied to calculate odd ratio (OR) and 95% confidence interval (CI) for evaluation of the association between MTHFR promoter methylation and oligozoospermia. When P<0.05, the correlation was considered statistically significant. All statistical analyses were performed using the SPSS v.20 software (IBM SPSS, USA).

### Computational analysis

An in silico analysis was performed using bioinformatics tools to evaluate the promoter features of *MTHFR*. Promoter and transcription start sites (TSSs) of *MTHFR* gene were diagnosed by Eukaryotic Promoter Database (http://epd.vital-it.ch) and DBTSS online software (http:// dbtss.hgc.jp), respectively. The presence of TATA boxes and PROSITE motifs were further investigated using MOTIF software (http://www.genome.jp/tools/motif). *MTHFR* CpG island, transcriptionally competent CpG islands (bona-fide CGIs), and nucleosome-exclusion regions were determined with UCSC Genome browser (http://genome.ucsc.edu/cgi-bin/hgTracks). Methylationsensitive TF binding sites (TFBS) in the regulatory region was predicted by the cisRED online server (http:// www.cisred.org). UCSC genome browser was used to characterize Cis-regulatory module (CRM). To detect TFs binding to CRM, this region was stretched using PReMod database (http://genomequebec.mcgill.ca/PReMod).

## Results

### Methylation specific polymerase chain reaction

By using the MS-PCR method we identified methylation patterns of *MTHFR* promoter region. After using the M-*MTHFR-f *and M-*MTHFR-r* primers in MS-PCR, a 186 bp fragment was detected on 8% polyacrylamide gel for methylated samples. Whereas, application of U-*MTHFR-f* and U-*MTHFR-r* primers demonstrated a 178-bp unmethylated fragment on the polyacrylamide gel. Therefore, the heterogeneous samples indicated both 186-bp and 178-bp fragments (Fig.1).

### *MTHFR*-CpG promoter methylation and oligozoospermia

The number of subjects was 151 individuals including 73 patients with oligozoospermia and 78 healthy controls. Characteristics of the oligozoospermia males and fertile controls are shown in Table 1. After MSP analysis, we found methylated (MM), unmethylated (UU) and heterogeneous (MU) status among the subjects. Frequency of methylated, unmethylated, and heterogeneous samples in patient were 8 (10.96%), 46 (63.01%), and 19 (26.03%), respectively; whereas, these numbers (ratios) in control group were 1 (1.28%), 68 (87.18%), and 9 (11.54%), respectively. The characteristics and semen parameters of 8 patients with hypermethylated *MTHFR* are depicted in Table 1. When we calculated Chi-square, a significant difference was found between case and control groups with regard to methylation status of *MTHFR* promoter. Additionally, OR in 95% CI showed a significant association between methylated (X^2^=7.98, OR=11.83, 95% CI=1.43-97.77, P=0.022) and heterogeneous (X^2^=6.85, OR=3.12, 95% CI=1.30-7.50, P=0.011) statuses of *MTHFR* promoter and oligozoospermia.

**Table 1:** Characteristics and sperm parameters of the subjects


Variables	Infertile		Fertile	P value*
	All	Methylated MTHFR	n=78	
	n=73	n=8		

Age (Y)	36.28 ± 3.49	34.25 ± 2.05	37.42 ± 4.11	0.069
Smoking (Y/N)	16/57	1/7	26/52	0.151
BMI (kg/m^2^)	23.41 ± 2.09	22.75 ± 2.12	24.00 ± 2.31	0.103
Seminal volume (mL)	3.19 ± 0.92	3.33 ± 0.95	3.26 ± 0.76	0.603
Sperm count (×10^6^/mL)	9.25 ± 3.17	9.38 ± 2.92	60.67 ± 10.10	<0.0001
Motility (% motile)	46.14 ± 7.25	47.13 ± 8.64	57.83 ± 8.88	<0.0001
Morphology (% normal)	41.41 ± 11.39	45.38 ± 9.30	51.95 ± 12.06	<0.0001


The data are expressed as mean ± standard deviation. *; The P value represents comparison of all infertile and fertile subjects.

### In silico analysis Description of *MTHFR* promoter

Three various promoter sequences were identified in *MTHFR* by Eukaryotic Promoter Database (EPD). There are four different *MTHFR* transcripts with different transcription start sites in the various tissues. Three polypeptides with 657, 698 and 680 residues are encoded by this gene. The data from EPD revealed that the promoter of *MTHFR* is near or in the CpG island. This shows the importance of epigenetic regulation of *MTHFR*. The CpG island with 1104 bp length has 93 CpG dinucleotide, 69.9% C or G, and 16.8% CpG. MOTIF software shows nine PROSITE motifs for the promoter region of *MTHFR* whereas there was not any TATA box in the region.

### Analysis of transcription-factor binding sites

ChIP-seq, cisRED and PReMod databases was used for analysis of TFBS in *MTHFR*. ChIP-seq server predicts position and architecture of TF in the genome. The data from this server revealed 44 TFs binding to the promoter region of *MTHFR*, 14 of which are related to spermatogenesis process (Table 2).

**Fig.2:**
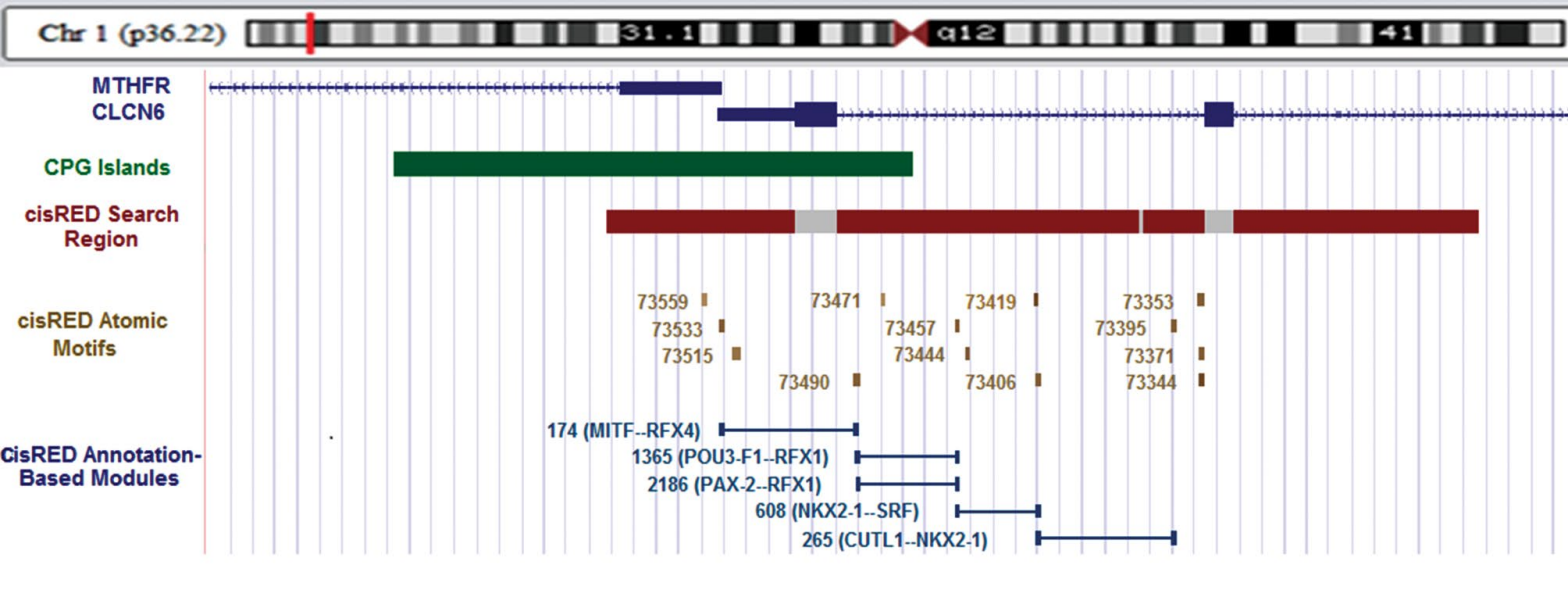
Location of cisRED atomic motifs and cisRED modules in MTHFR promoter. The long green bar at the top shows location of CpG island. The red and gray bars indicate nominal ’search region’ within which comparative genomics discovery methods were applied by cisRED. The numbered brown blocks are ‘atomic’ motifs, i.e. conserved DNA sequence motifs that were identified by discovery methods and post-processing operations. Blue lines show annotation-based modules.

**Fig.3:**
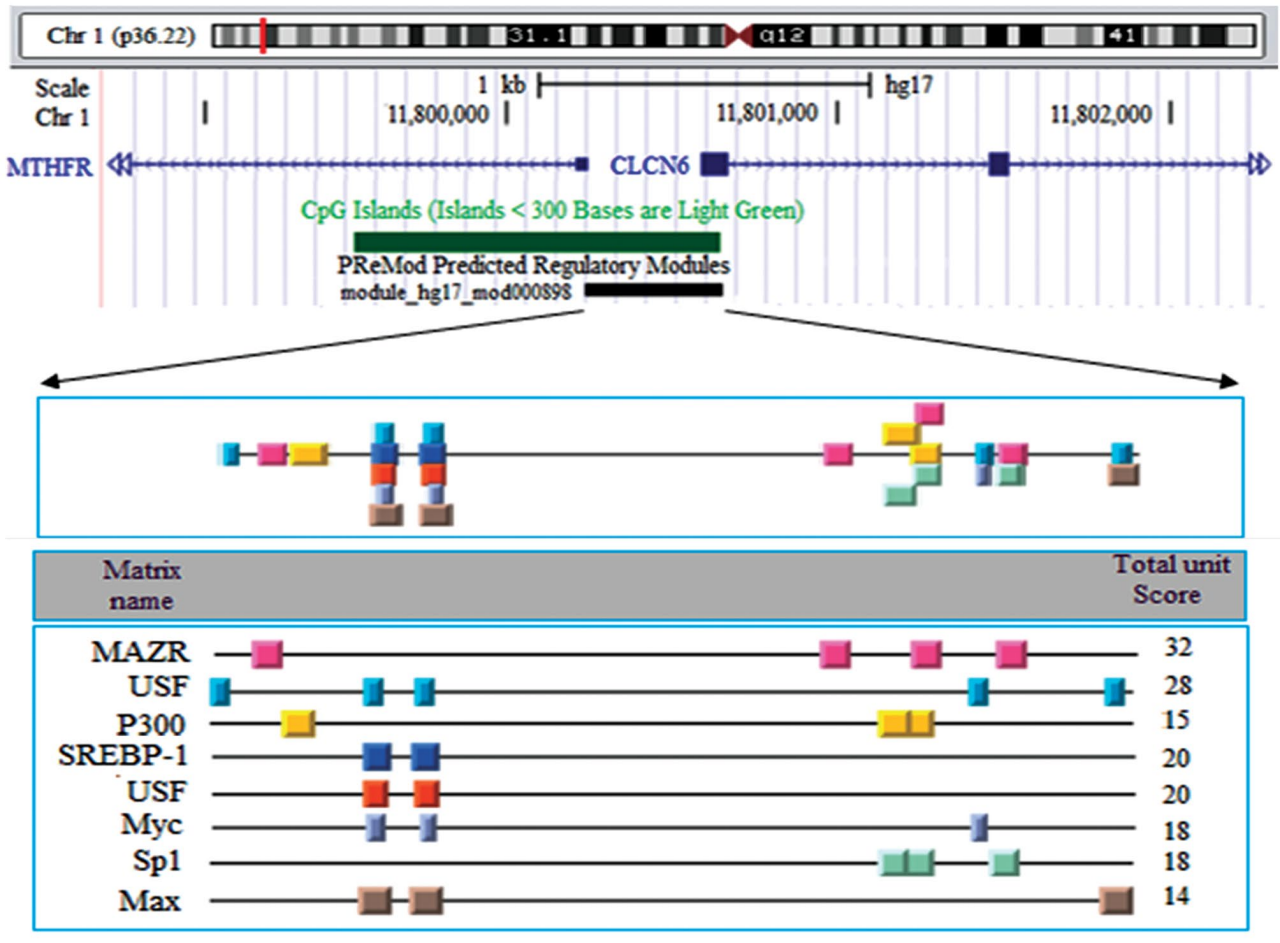
The module mod000898 and related transcription factors predicted by PReMod database. The black bar shows exact position of this module in CpG island and the precise location of transcription factor binding sites in this module is demonstrated by colorful boxes.

The data obtained from cisRED program showed that 13 atomic motifs, as an evolutionary conserved sequence recognized in the promoter region via a comparative examination of more than 40 vertebrate species and five annotation based modules, exist in *MTHFR* regulatory region. According to this database, *MTHFR* regulatory region with 1854 bp expands at the 5´ end of CpG island (Fig.2). The module mod000898 in *MTHFR* promoter, predicted by PReMod database, overlaps with 5´ end of CpG island. This module (mod000898) with 418 bp size has score 460 and it exactly overlaps with the amplified fragment in our research. Moreover, the PReMod server showed that Sp1, MAZR and p300 are the most important TFs which regulate *MTHFR* expression (Fig.3).

### Bona fide CpG island

**Fig.4:**
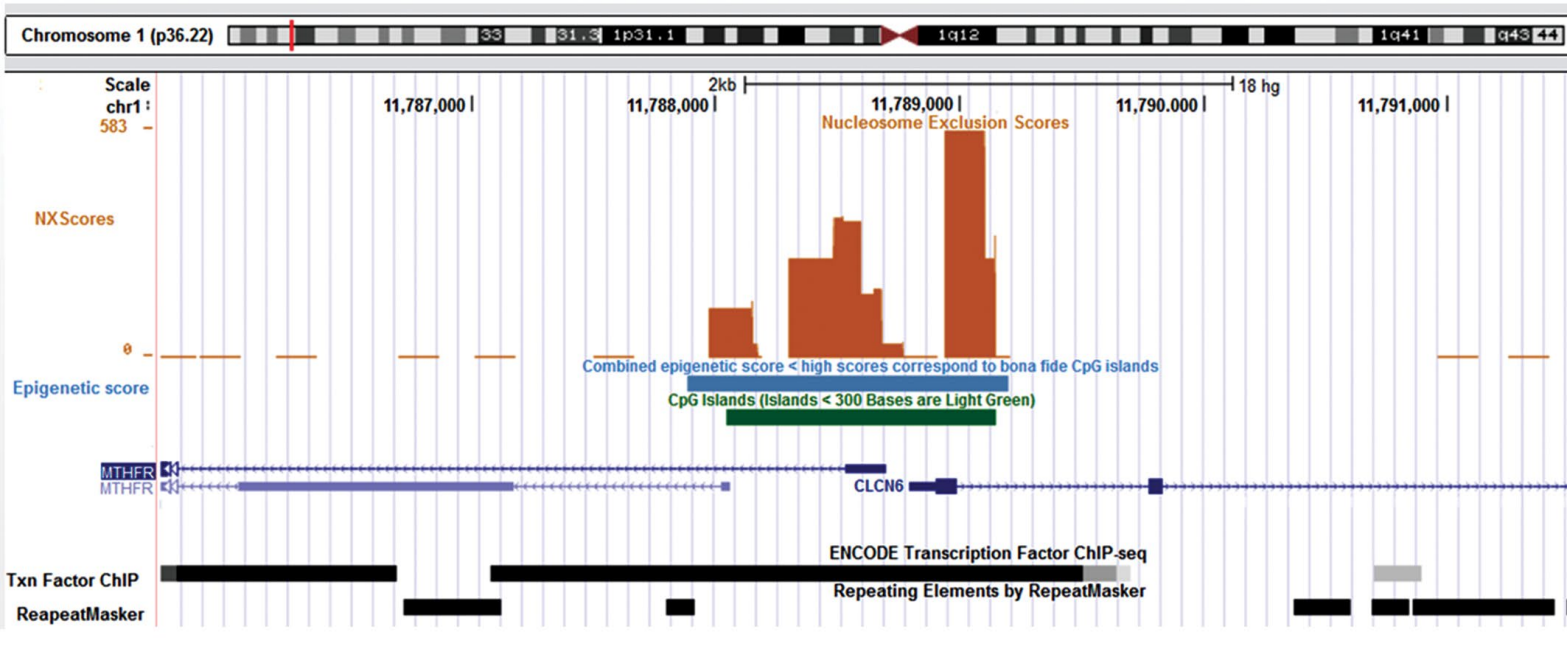
CpG Island, bona fide and nucleosome exclusion score (NXScore) of *MTHFR* promoter. CpG island expands in 5’ end of* MTHFR* gene and even beyond it. Bona fide area overlaps with and even broader than CpG island. The NXScore graph implicates low density of nucleosome at the promoter region of *MTHFR*.

We detected “bona-fide CGIs”, a high accuracy method to identify CpG islands by integrating their genetic and epigenetic features, for *MTHFR* gene. Bona-fide CGIs that introduces epigenetic and genetic states (such as open chromatin structure, frequent promoter activity, euchromatin, DNA helix structure, significant conservation and repeat depletion) was detected at the promoter region of *MTHFR* with the score 573 (Fig.4). As depicted in Figure 4, the bona fide CGIs has not only positional overlap with CpG island, but also even with a larger length than CpG island.

### Nucleosome exclusion region

Nucleosome exclusion regions (NXRegions) were predicted by a custom track in UCSC genome browser. The data from this server revealed that there are three regions with the high score of NXRegions in the promoter of *MTHFR* (Fig.4). In addition, there were some transcription start sites in these NXRegions.

**Table 2:** Transcription factor binding site in *MTHFR* promoter involved in spermatogenesis process


Factor	Size	Score	Target description

NFKB	88	139	NFKB acts as a regulator TF in the Sertoli cell-spermatid junctional complexes. In the spermatogenesis processes, selenium in corporate with NFKB has a critical regulator function.
STAT1	1710	149	STAT1 as a member of STAT family has an important role in development, prevention of proliferation and immune response. It acts as a regulator which controls the gene transcription in Sertoli cells
AP1	1792	182	This factor is required through the G1 phase of the cell cycle
c-Myc	2314	386	It acts as a multifunctional protein which has roles in cell cycle, apoptosis and cellular transformation
Max	1926	1000	This factor with other families such as Myc which is an oncoprotein involved in cell proliferation, differentiation and apoptosis
FOSL2	1144	1000	FOS family applies as regulators of cell proliferation, differentiation, and transformation
GABP	1013	886	GA-binding protein (GABP) acts as a regulator of gene expression. It regulates some crucial genes which incorporate in cell cycle, protein synthesis, and cellular metabolism
p300	962	1000	This factor regulates transcription by chromatin remodeling, and it involves in cell proliferation and differentiation
PAX5	740	807	Expression of this factor has been identified in the developing testis, implicates its role in spermatogenesis
SP1	917	832	This well-known factor involved in many cellular processes such as cell development, differentiation, immune defense, apoptosis, chromatin remodeling and response to DNA damage
POU2F2	754	424	This protein has multiple functions such as immune response, embryogenesis, neurogenesis, and etc
Sin3Ak-20	772	1000	This protein with histone deacetylases (HDACs) manages gene silencing. Sin3/HDAC is also involved in genomic stability, cell cycle development, embryonic progression, and homeostasis
BHLHE40	274	345	BHLHE40 roles as a transcriptional repressor. It controls cellular progression, development and differentiation
USF-1	216	71	USF1 with p53 takes part in cell fate decisions. It also simplifies the switch of proliferation to differentiation of sertoli cells in testes


## Discussion

In this study, we investigated association of *MTHFR* promoter methylation with oligozoospermia in an Iranian population. Our results indicated that there was a significant association between the promoter methylation and oligozoospermia. Consistent findings were also reported in some previous studies. Houshdaran et al. (17) suggested a significant association between abnormal semen parameters and hypermethylation in several genes. They indicated that improper erasure of DNA methylation during epigenetic reprogramming would be the cause of disorder. In the other study, Wu et al. (18) reported hypermethylation of *MTHFR* promoter in sperms and its association with idiopathic male infertility. Moreover, Khazamipour et al. (14) detected hypermethylation of *MTHFR* promoter region in testis biopsies. They reported that more than half of the patients with non-obstructive azoospermia (NOA) were hypermethylated in the *MTHFR* promoter, while the patients with obstructive azoospermia had a normal methylation pattern. Botezatu et al. (19) also reported that *MTHFR* hypermethylation may be associated with male infertility in Romanian population. Rotondo et al. (20) observed a correlation of hypermethylation status of *MTHFR* promoter in semen samples of 55% of infertile couples with recurrent spontaneous abortion (RSA), compared to 8% in non-RSA (NRSA) and 0% in fertile couples. They suggested *MTHFR* methylation as a novel putative risk factor for RSA etiology. All of these studies proved the important role of epigenetic regulation of *MTHFR* in male infertility. However, the geographical, racial and environmental factors may modulate the effects of *MTHFR* promoter methylation

The testis of an adult male is composed of more than 80% germ cells, chiefly in the meiotic and post-meiotic phases of spermatogenesis (21). Some studies presented the patterns of DNA methylation in different cells throughout spermatogenesis. Oakes et al. (22) mentioned that testicular germ cells established their pattern of methylation genome prior to meiosis, first of all in spermatogonia and after that in early prophase I spermatocytes. Another study reported that DNA methylation is maintained steadily during spermatogenesis and spermiogenesis (6). Farthing et al. (13) evaluated DNA methylation of 26,275 promoters and indicated that the patterns of methylation with hypomethylation states were similar in embryonic stem cells, embryonic germ cells and sperms. Therefore, based on the mentioned previous study, aberrant DNA methylation pattern of spermatozoa from infertile men reflects methylation status of adult germ cells throughout the seminiferous tubules. In this regards, Rotondo et al. (20) reported that *MTHFR* hypermethylation detected in spermatozoa samples could be representative of the status of adult germinal stem cells.

Folate metabolism has a critical role in DNA synthesis, DNA protection and epigenetic modifications such as histone or DNA methylation. *MTHFR* is one of the key enzymes which regulate folate metabolism. Dysfunction of the enzyme leads to the adverse effect on spermatogenesis and it may result in infertility. *MTHFR* expression can be regulated by genetic and epigenetic factors. Here, we investigated the role of *MTHFR* promoter methylation as an epigenetic factor in oligozoospermia, followed by computational analyses. We detected an altered pattern of promoter methylation in the oligozoospermic man compared to healthy controls. Our data revealed that there is a significant association between methylated CpG islands in *MTHFR* promoter region and oligozoospermia. Hypermethylation of this area causes down-regulation in *MTHFR* gene expression. Therefore, it causes folate pathway defect followed by some adverse results, such as homocysteine accumulation, DNA impairment, genome destabilization, defects in sperm proliferation and differentiation. All of these abnormalities may result in oligozoospermia.

Genetic and epigenetic variations may affect gene expression and bioinformatics is a useful tool for evaluation of these effects (23, 24). In this study we applied a bioinformatics approach to investigate effects of *MTHFR* promoter methylation in regulating gene expression. Our research revealed strong evidence for epigenetic regulation of the *MTHFR* promoter. For example, we detected the NXRegion in this promoter. Nucleosomes are one of the basic structures for DNA condensation and they contribute to epigenetic regulation of genes (25). Their interaction with DNA leads to chromatin condensation, whereas promoter region in active genes are nucleosome free, so that TFs can bind to DNA and start the transcriptional event (26). CpG dinucleotides in DNA are one of the factors which modify strength and condensation of nucleosomes (27). Methylation of CpG dinucleotide could directly affect DNA bendability and nucleosome positioning (28). There is a reliable association between tissue specificity of a coding sequence and tendency of its promoter to exclude nucleosomes. Some computational algorithm predicts in vivo nucleosome positioning through a specific gene. Here, we used a custom track which shows NXRegions in the genome sequence. In this software nucleosome exclusion score for *MTHFR* gene was positively correlated with gene expression levels and DNase I hypersensitive sites (29). Peaks in graph generally are associated with transcription start sites. Based on MOTIF software, there is six PROSIT motif in the promoter region of *MTHFR*, but there was not any TATA box in the region. Absence of TATA box in this promoter suggests that it is less conserved compared to a common promoter. The “bona-fide CGIs”, is a marker with a high accuracy to identify CpG islands by integrating their genetic and epigenetic features. This marker identified this region in *MTHFR* promoter. The length of this area is a determining parameter to ensure that epigenetic properties of a fragment are not missed (30). Furthermore, the promoter region of *MTHFR* is an area which binds to several TFs, 14 of which are directly involved in fertility process.

## Conclusion

Based on our findings, promoter methylation of *MTHFR* gene may be a risk factor for oligozoospermia. However, further studies with a larger sample size among different ethnicity are needed to obtain more accurate results. There were some limitations in our study which should be considered. Firstly, the study suffered small sample size for the case and control groups. We also did not evaluate the effects of methylation on gene expression. Furthermore, the influence of environmental factors such as folate intake was not assessed.
